# Electronic portfolio use in pediatric residency and perceived efficacy as a tool for teaching lifelong learning

**DOI:** 10.1186/s12909-017-1045-6

**Published:** 2017-11-10

**Authors:** Annabel Frank, Kimberly Gifford

**Affiliations:** 1 Perelman School of Medicine at the University of Pennsylvania, Expected M.D. 2018, 1 Bluxome Street, Suite 205, San Francisco, CA 94107 USA; 2 Geisel School of Medicine at Dartmouth, Program Director, Children’s Hospital at Dartmouth-Hitchcock Pediatric Residency, One Medical Center Drive, Lebanon, NH 03766 USA

**Keywords:** Electronic portfolio, Lifelong learning, Individualized curriculum, Self-reflection, Goal setting, Competency

## Abstract

**Background:**

Residency programs use electronic portfolios (efolios) to organize data, track resident performance, and sometimes teach and assess lifelong learning (LLL) skills. Published studies on efolios in graduate medical education are mostly descriptions of implementation at individual institutions.

**Methods:**

An anonymous online survey was sent to 199 pediatric residency program directors across the United States. Efolio usage patterns were described and compared between program directors that perceived efolios effective at fostering LLL and those that did not.

**Results:**

Surveys were completed by 82 of 199 program directors (41%), and 55% used efolios. The 20% (9 of 45) of program directors that believed efolios were effective at teaching LLL more often used self-assessment (88% vs. 50%, *p* = 0.05) and goal-setting (75% vs. 40%, *p* = 0.03) functionalities. Common efolio challenges included limited usability and difficulty integrating data. Most non-users (65%) would like to invest in efolios.

**Conclusions:**

Respondents reported technical and convenience-related challenges to efolio use, which need to be addressed for efolios to meet their potential as valuable learning tools. The use of self-assessments and goal-setting features was associated with program directors’ perceptions that efolios were effective at fostering LLL.

## Background

An electronic portfolio (efolio) is a web-based, centralized electronic system that supports the management of personal information, professional and educational accomplishments, and career development plans [1]. The most fundamental efolio function is data organization. Many systems also allow information sharing. Some systems enable learners to reflect on their experiences and set goals for the future [1]. Due to the lack of commercial efolios with the needed functionalities, some programs that can afford the investment have created their own systems [2–4]. Individual programs at all levels of education have examined the factors that influence efolio success in supporting medical training. Systematic reviews describe several factors contributing to success including: integration with curriculum and assessment, support through mentoring, and measures to reduce time demands [5, 6].

Efolios are being developed to better support teaching, assessing, and tracking the development of competence. Efolios may play an even more important role for pediatric residents in the United States, given the new requirement for 6 months of training individualized to their learning needs and career plans. As more training programs worldwide move beyond data synthesis and use efolios to teach complex competencies, it is important to identify which factors promote these outcomes. Institutions that have studied the implementation of efolio curricula with emphasis on mentorship [2, 3, 7–9] and protected time [2, 7, 8] have reported success in promoting lifelong learning (LLL) skills. Practicing reflection, self-assessment, and goal setting may be easier for learners who have the ability to view their external assessments, identify areas for improvement, and track their progress over time in an efolio. Current research on efolios in residency consists primarily of single institutions discussing their experiences implementing a system. Single institution studies like these provide insight into how efolios might function as valuable organizational and education tools; however, many program directors (PDs) report implementation challenges as learners find the systems time-consuming and frustrating [3, 10–12]. Further, each intervention is context-specific and the conclusions from one institution may not be generalizable.

The purpose of our study was to examine how efolios are used across all pediatric programs. We aimed to assess functionalities used, perceptions of efficacy for LLL, communication through efolios, barriers to use, interest in future use, and access after residency. We were especially interested in which features may support LLL and hypothesized that the use of reflection, self-assessment, identifying areas for improvement, and goal setting would be associated with the perception of efolios as LLL tools.

## Methods

We developed an electronic survey to assess efolio perceptions and patterns of use. Content validity was established through examination of the efolio literature and review of the survey by the Association of American Medical Colleges (AAMC) efolio advisory board. Response process and construct validity were examined by piloting the survey with a group of PDs and asking for their feedback on the content, explanations, and format. Results of the pilot led to modification of the instructions to include a definition of electronic portfolios and the addition of answer options suggested by free text responses to “other” answer choices. The survey was exempted from review by the Center for the Protection of Human Subjects at Dartmouth and approved by review processes at the Association of Pediatric Program Directors (APPD) and the AAMC. A link to the anonymous survey was emailed by the APPD administration in the summer of 2013 to the 199 pediatric PD members with two follow-up reminder emails. The survey consisted of 20 multiple-choice and 5 open-ended questions [13]. Questions were different depending on whether a respondent indicated their institution used an efolio system or not, but the length of survey was the same. It was designed to take 5–15 min. The distribution of responses was tabulated for each survey item. Responses were grouped according to whether PDs believed their efolios were *effective* (including “somewhat effective” and “very effective” responses) or *not effective* (including “neutral,” “somewhat ineffective,” and “very ineffective” responses) in creating a learner-centered curriculum and teaching LLL. We compared the usage rate of each efolio feature between the *effective* and *not effective* groups using one-sided Fisher’s exact test. We analyzed the qualitative responses using inductive content analysis [14]. AF used open coding to organize responses into codes. KG reviewed the coding for agreement and codes were grouped into themes.

## Results

### Current use and perceptions

The survey was completed by 82 of 199 PDs (41% response rate). Of the 82 respondents, 71 (87%) answered at least one open-ended question. Respondents included 79 PDs, 2 associate PDs, and 1 coordinator. Overall, 55% (45 of 82) of respondents used efolios. A greater proportion of medium-sized programs (30–60 residents) used efolios (73%; 27/37) than those smaller (43%; 10/23) and larger (36%; 8/22) programs. The distribution of program sizes for respondents (28% small, 45% medium, and 27% large) was similar to the national distribution (25%, 47%, and 28%). Resident use was required by 32 of 45 programs (71%). Most PDs reported residents used the systems < 1 or 1–2 h per month (Table 1). Two PDs reported residents used the systems > 6 h per month.

**Table 1:** Time Residents Spent Using Efolios as Reported by Programs (*n* = 45)

Average hours per month	Programs Reported Resident Use No. (%)
< 1 h	17 (38)
1–2 h	14 (31)
3–5 h	2 (4)
6–10 h	1 (2)
> 10 h	1 (2)
No response	10 (22)

Efolio features were used by the majority of programs (≥ 74%) for management and synthesis of biographical information, case/procedure logs, resident performance reports, and program evaluations (Table 2). Least frequently used functions included recording reflections (26%), recording extracurricular activities (24%), creating CV/resumes (8%), and group work (3%).

**Table 2:** Efolio Functionalities Used as Reported by Program Directors (*n* = 38)

	Total Programs Using Feature No. (%)	“Effective Subgroup”^b^ Using Feature No. (%)	“Not Effective”^c^Subgroup Using Feature No. (%)	*P* value^d^
	(*N* = 38)	(*N* = 8)	(*N* = 30)	
Manage personal profile (bio information)	33 (87)	8 (100)	25 (83)	NS
Case or procedure logs	28 (74)	6 (75)	22 (73)	NS
Synthesize information for resident performance reports	28 (74)	7 (88)	21 (70)	NS
Compile information for program evaluation and reports	28 (74)	7 (88)	21 (70)	NS
Identify areas of improvement	23 (61)	7 (88)	16 (53)	NS
Self assessment (inventories, competency skill-tracking, etc.)	22 (58)	7 (88)	15 (50)	*0.05
Manage documents (presentations, publications, etc.)	19 (50)	3 (38)	16 (53)	NS
Create and track personal and professional goals	18 (47)	6 (75)	12 (40)	*0.03
Benchmark resident performance with other residents	18 (47)	5 (63)	13 (43)	NS
Record personal or professional reflections (free-write, journaling, etc.)	10 (26)	4 (50)	6 (20)	NS
Record ‘other’ extracurricular activities (volunteering, clinical work, etc.)	9 (24)	2 (25)	7 (23)	NS
Create CV/Resume template	3 (8)	1 (13)	2 (7)	NS
Collaborate - facilitate group work	1 (3)	1 (13)	0	NS

^a^The n value excludes missing cases because of item nonresponse

^b^Subgroup that reported their efolios were *effective* (including “somewhat effective” and “very effective”) at teaching LLL

^c^Subgroup that reported their efolios were *not effective* (including “neutral,” “somewhat ineffective,” and “very ineffective”) at teaching LLL

^d^From Fisher’s exact test. Programs were asked to report if feature was utilized, available but not utilized, would be nice, or not important. Comparison was made between programs that responded “utilized” and those that responded “available but not utilized” or “not important”

Some PDs considered efolios effective at tracking development of competence (47%; 21/45), creating a learner-centered curriculum (31%; 14/45), and teaching LLL skills (20%; 9/45). A subgroup analysis revealed PDs that believed their efolios were effective at teaching LLL skills used the systems in distinctive ways (Table 2). Compared to PDs that did not consider the efolios effective, a greater proportion of this subgroup reported residents used efolios to create and track personal and professional goals (75% vs 40%, *p* = 0.03) and for self-assessments (88% vs 50%, *p* = 0.05). Other trends in usage by this subgroup included more frequent use of benchmarking performance (63% vs 43%, *p* = 0.07), identifying areas of improvement (88% vs 53%, *p* = 0.09), and reflections (50% vs 20%, *p* = 0.13).

### Communication through Efolios

Few PDs reported efolios were the preferred method of communication about education (11%, 5 of 45) and administrative tasks (20%, 9 of 45). Respondents cited the convenience of centralized reporting and evaluation. Comments on cultural barriers to communicating through efolios indicated residents check email and text more regularly. Responses included descriptions of the systems as “cumbersome,” “not user-friendly,” and “impersonal and inefficient”.

### Barriers to use

Respondents were asked about concerns expressed by staff and residents using efolios (Table 3). The top complaints were technical: limited customizability, usability, and functionality and difficulty collecting, importing, and exporting data. One PD commented that many features were not used because “system not sophisticated enough to use well.” The frequency of complaints was the same amongst PDs that thought efolios were effective at promoting LLL and those that did not.

**Table 3:** Complaints Expressed by Residents and Staff Using Efolios as Reported by Programs (*n* = 45)

	Programs Reporting Complaints No. (%)
Collecting, importing, and exporting data	21 (47)
Limited Customizability	20 (44)
Limited time (personal or professional) to use the system	19 (42)
Usability	19 (42)
Limited functionality	17 (38)
No access after residency	15 (33)
Organization	14 (31)
No mobile access	11 (24)
Technical support	8 (18)
Case or procedure log limitations	7 (16)

### Desired Efolio features

Most PDs (65%; 24/37) that did not currently use an efolio were interested in investing and several were transitioning. When asked their reasons for investing, most commented that efolios might assist in the required tracking of resident milestone progression. PDs not interested in efolios expressed concerns that the expense would not be worthwhile given that an inefficient system could go unused or be time-consuming and ineffective.

PDs interested in efolio investment completed questions about potential systems. All or nearly all (96%; 23/24) believed the following features would be important: ease of use, customizable, import/export of information (biographical, certification, licensing), document management, and creation of performance reports and CVs. Features considered important to fewer PDs, but still the majority, included collaboration (79%; 19/24) and reflections (71%; 17 of 24).

### Access after residency and LLL

Nearly all non-users (92%; 22/24) would want residents to have efolio access after residency. However, only 13% (6/45) of PDs using efolios reported their residents maintain access. When asked why access after residency would be important, non-users discussed ease of access to information, particularly for privileging and ongoing accreditation. Non-users also commented on the importance of access for continuation of learning goals, reflection, and LLL.

Several PDs using efolios (13%; 6/45) reported residents have requested access after graduation, primarily for procedure logs. Responses to why residents do not request access included: “used very little during residency,” “use isn’t fully inculcated,” and “not that useful.” Comments indicated some residents download information before leaving. One program reported residents have to pay to maintain access after graduation. No PDs were willing to pay for continued access.

## Discussion

This is the first study of efolio use across a national sample of Pediatric residency programs. The results substantiated many concerns expressed in studies of individual residency programs [3, 10–12]. PDs place high value on ease of data management and synthesis, but most report that their current efolio systems do not meet these needs. Information management is integral to efolios, yet the most commonly reported complaint was difficulty collecting, importing, and exporting data. PDs that have yet to adopt efolios are concerned that the systems may lead to wasted time and money without sufficient compensatory benefits*.*

While 92% of non-users reported that self-assessment and goal tracking would be important features, fewer PDs with efolios take advantage of them: 58% use self-assessment and 47% use goal tracking. This gap between desire and actual utilization suggests that current systems are not being used in the ways that institutions might have hoped or intended. The challenge of getting residents and faculty to embrace efolios has led to various interventions to improve their acceptance and use [3, 8, 15]. Recommendations from other studies regarding integration include: educating residents and faculty about the goals and importance of the system [8], protected time for use [2, 7, 8], and faculty feedback and mentorship [2, 3, 5–9, 16, 17]. Providing protected time or compensation for both residents and advisors to engage in meaningful efolio use may also promote LLL [2, 7, 8].

The individualized curriculum in Pediatric Residency provides an opportunity for protected time and mentorship for residents to focus on their learning needs and career plans. Thus, Pediatric residency programs are in a unique position to use portfolios as a tool to promote LLL.

Adult learning theory emphasizes the importance of experience, and technology has the potential to capture experiences. Well-designed apps encourage people to document and share, for example Twitter, Facebook, Instagram, and blogs. These programs are user-friendly and make it fun and rewarding to keep a sort of electronic diary. Efolios can provide similar features. With functions for recording, sharing, getting feedback, discussing, and reviewing, efolios could provide an individualized approach to lifelong learning.

Some of the major limitations to this study are that it was based on PD perceptions rather than other measures of efolio efficacy or resident perspectives. The response rate was only 41% and non-respondents may have different patterns of efolio utilization and opinions. We only sampled Pediatric Residency Programs in the United States; however, the push to use efolios for teaching and assessment and the need for convenient data systems are shared by graduate medical education programs around the world and across a variety of specialties [2, 4, 6, 11, 12, 15]. It is possible that the way efolio functionalities are used is unique to the institution; therefore, it is hard to draw conclusions from simply which functionalities are used.

Future studies should investigate how efolios are incorporated into resident training across programs. Resident and faculty perspectives should be obtained through interviews and surveys. As better methods to assess resident LLL are created, the impact of efolio usage on resident outcomes should also be examined.

## Conclusion

The results of this study suggest that the technological design of efolios should be improved to better meet organizational needs such as data synthesis and organization. PDs would appreciate systems that are more user-friendly, convenient, and integrated. The potential of efolios to facilitate LLL skills may be related to how they are used. It is possible that improved technology could promote efolio use for activities that promote LLL such as reflection, self-assessment, and goal setting.

## Abbreviations

AAMC: Association of American Medical Colleges
APPD: Association of Pediatric Program Directors
Efolio: Electronic portfolio
LLL: Lifelong learning
PD: Program director
